# Brain-Derived Neurotrophic Factor Levels in Mesenchymal Stem Cell Culture Fluid Under Different Culture Conditions

**DOI:** 10.5152/eurasianjmed.2021.0138

**Published:** 2023-02-01

**Authors:** Gülsemin Çiçek, Muharrem Çiçek, Emine Utlu Özen, T.Murad Aktan, Faik Özdengül, Selçuk Duman

**Affiliations:** 1 Department of Histology and Embryology, Başakşehir Çam and Sakura City Hospital, İstanbul, Turkey; 2 Department of Pediatrics, Kanuni Sultan Süleyman Training and Research Hospital, İstanbul, Turkey; 3 Department of Histology and Embryology, Etlik Zübeyde Hanim Women’s Health Training and Research Hospital, Ankara, Turkey; 4 Department of Histology and Embryology, Necmettin Erbakan University, Meram Faculty of Medicine, Konya, Turkey; 5 Department of Physiology, Necmettin Erbakan University, Meram Faculty of Medicine, Konya, Turkey; 6 Department of Histology and Embryology, Necmettin Erbakan University, Meram Faculty of Medicine, Konya, Turkey

**Keywords:** Mesenchymal stem cell, culture fluid, brain-derived neurotrophic factor, hypoxia, microcarrier

## Abstract

**Objective:**

Mesenchymal stem cells are used in various fields, such as cellular therapy, regeneration, or tissue engineering. It has been shown that they exhibit many protective factors and also work as a modulating chief within the region in which they are administered. There are studies on both the therapeutic and neuroprotective effects of brain-derived neurotrophic factor. Also, there are many studies on the improvement of culture conditions for in vitro reproduction of mesenchymal stem cells, which can be obtained from many sources in various bodies, such as adipose tissue and Wharton’s jelly. Improving and standardizing these culture conditions will increase the effectiveness and reliability of stem cell therapies. Studies evaluating many culture conditions, such as O_2_ level, type of medium, monolayer culture, and the transition from in vitro 3D models, are ongoing.

**Materials and Methods:**

In our study, groups were formed by using stem cells originating from adipose tissue and Wharton’s jelly. Stem cell cultures were made using Hillex-II and Pronectin-F microcarriers. Cell culture O_2_ level was adjusted as 1% and 5% for each group separately. Enzyme-linked immunosorbent assay was used to analyze brain-derived neurotrophic factor levels in stem cell culture fluid.

**Results:**

The highest brain-derived neurotrophic factor level in mesenchymal stem cells culture medium was observed in an adipose-derived stem cell culture with an in vitro fertilization (non-treated) dish, using a Hillex microcarrier in a 1% O_2_ microenvironment.

**Conclusion:**

As a result of our observations, we think that cells could exhibit greater therapeutic potential in a dynamic adhesion environment.

**Main Points**

- Stem cell-based treatments are used more and more every day in the clinic. Besides the stem cells themselves, the therapeutic potential of the factors released into the culture medium in which they are located is among the current issues.
- Mesenchymal stem cells exhibit many protective factors and also work as a modulation chief in the area where they are applied.
- Many factors such as temperature, oxygen level, and culture medium contents and the effects of the mechanical environment of mesenchymal stem cells grown in vitro are the subjects that continue to be investigated. Instead of traditional 2-dimensional (2D) culture conditions, 3D dynamic conditions began to take.
- Brain-derived neurotrophic factor has neuroprotective effects in the treatment of neuron damage and there are still experimental studies on this subject.
- In the future, it is inevitable that innovations will emerge and provide the most optimal conditions approaching the in vivo environment of cells.

## Introduction

The method of cell culture is one of the important tools used in cellular and molecular biology for understanding physiology and biochemistry of cells, including intracellular activity (e.g., protein synthesis, drug metabolism), environmental interaction (e.g., nutrition, cytotoxicity, drug action, carcinogenesis), cell–cell interaction (e.g., paracrine control, morphogenesis, cell adhesion, and motility), cell products, and secretion.^1^

The 2 cell culture systems used today are traditional 2-dimensional (2D) and 3-dimensional (3D) cell cultures. Two-dimensional cell culture is one of the most common cell culture techniques used routinely in laboratories worldwide.^2^ However, this traditional monolayer culture method is not an optimal in vivo model due to the loss of characteristic cell morphology and in vivo microenvironmental conditions during passaging in addition to the alteration of cell-specific extracellular matrix secretions.^3,4^ Therefore, this condition has many disadvantages, such as a significant negative impact on cell performance and the results of biological assays.^5^ Compared with 2D monolayer cell cultures, 3D models have a particularly important role in modern cell biology. Three-dimensional models provide suitable microenvironments for optimal cell growth, differentiation, and function, mimicking the complex cellular interactions closer to in vivo conditions.^5-7^ The various 3D culture models, such as whole animals and organotypic explant cultures, microcarrier cultures, cell spheroids, and tissue-engineered models, can be used for studies.^2^

Microcarrier technology was first used by van Wezel in 1967.^8^ Microcarrier beads, coated with various synthetic and natural (collagen, gelatin, etc.) polymers to increase cell attachment, provide a large surface area to culture large numbers of cells in small volumes.^3,4,7^ Microcarrier surface area, size, porosity, material nature, and functional attachment groups are also important factors that affect cell adhesion, proliferation, and differentiation.^5^

Stem cells—especially mesenchymal stem cells (MSCs)—are widely used in stem cell therapy; therefore, they are currently one of the most popular research areas.^9^ Mesenchymal stem cells have been isolated from bone marrow, adipose tissue, peripheral blood, Wharton’s jelly of the umbilical cord, and other tissues.^10^ Mesenchymal stem cells differentiate into several terminally differentiated cell types, such as osteoblasts, chondrocytes, adipocytes, hepatocytes, and neuronal and glial lineages.^11^ Studies have shown regeneration, tissue repair, and immunomodulation capacities of MSCs. Secretomes are thought to be responsible for the beneficial effects of MSCs in degenerative and inflammatory diseases of the nervous, gastrointestinal, respiratory, musculoskeletal, and cardiovascular systems.^12,13^

Brain-derived neurotrophic factor (BDNF) is expressed in the central and peripheral nervous system and non-neuronal tissues. It plays an important role in neurogenesis, neuronal development, neuronal plasticity, synaptic function, and other processes, such as cancer and angiogenesis.^14^ In literature, several studies have shown that MSCs produce growth factors such as BDNF,^15^ nerve growth factor, hepatocyte growth factor, and vascular endothelial growth factors.^11^

Studies have shown that transplanting MSCs after hypoxic preconditioning is an effective way to increase the regenerative capacities and therapeutic potential in ischemic stroke treatment, compared to normoxic conditions.^16,17^

In our study, the highest BDNF level in MSC culture medium was observed in an adipose-derived stem cell culture with an in vitro fertilization (IVF) (non-treated) dish, using a Hillex microcarrier in a 1% O_2_ microenvironment.

## Materials and Methods

This study was approved by the Research Ethics Committee in accordance with the World Medical Association Declaration of Helsinki (approval number: 14567952).

## Adipose Tissue-Derived and Wharton’s Jelly-Derived Mesenchymal Stem Cell Cultivation

### Isolation of Mesenchymal Stem Cells

**Adipose Tissue-Derived MSC: **Liposuction aspirates from subcutaneous adipose tissue sites (abdomen, flank, and thighs) were obtained from female subjects undergoing elective plastic surgical procedures. All donors gave their written informed consent. After 10-20 g of fat material was washed with saline, it was treated with 0.1% collagenase enzyme at room temperature, and the components were washed. Mesenchymal stem cells were observed in the supernatant after centrifugation at a speed of 300-500 g. Cell culture consisted of Dulbecco’s modified Eagle medium (DMEM) with additives of 7.5% platelet lysate, penicillin–streptomycin, and l-glutamine at 37°C.

**Wharton’s Jelly-Derived MSC: **A purchased cell line (ATTC PCS 500-010, USA) was used. Cell culture consisted of DMEM with additives of 7.5% platelet lysate, penicillin–streptomycin, and l-glutamine at 37°C.

### Stem Cell Characterization and Multipotency

**Figure 1. A,B.**
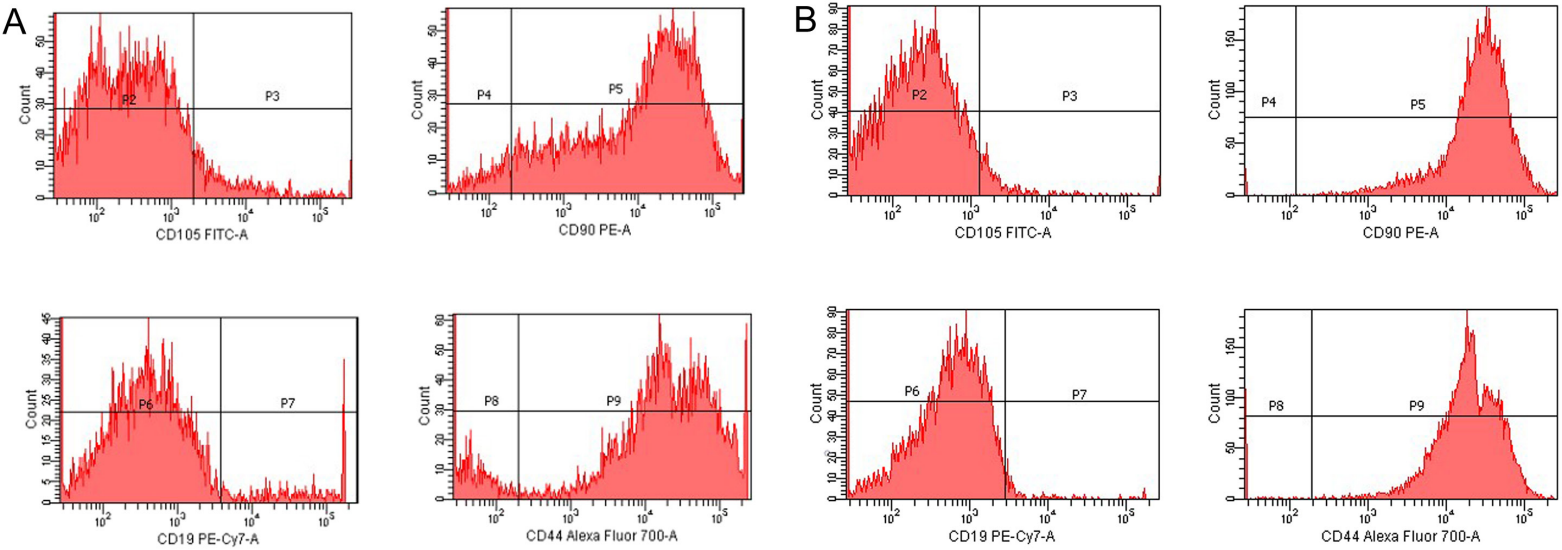
Characterization of Wharton’s jelly-derived stem cell (A) and adipose tissue-derived stem cell (B) by flow cytometry (analysis of CD90, CD44, CD105, and CD19 cell surface markers).

**Flow cytometry: **CD44, CD90, CD73, and CD19 cell surface antigens (Human Mesenchymal Stem Cell Flow Cytometry Kit, Novus Biologicals, Littleton, CO, USA) for both cell groups on 3D cell culture were analyzed for stem cell characterization with flow cytometry (FACSDiva Version 6.1.3) (Figure 1).

Differentiation: adipogenic, osteogenic, and chondrogenic differentiation capacity of mesenchymal stem cells were studied^9^

**Adipogenesis Assay:** Cells were seeded in 96-well plates (3 × 10^2^ cells/cm^2^). After 24 hours, it was ascertained that they had reached approximately 80% confluence, and the medium was replaced with an adipogenic differentiation medium (MSCgo Adipogenic XF, Biological Industries, USA), in which they were incubated for 21 days. Subsequently, they were fixed in 10% formalin at room temperature for 30 minutes. They were then incubated in 60% isopropanol and Oil Red O working solution. Lipid accumulation was visualized as lipid-containing red droplets using light microscopy.

**Osteogenesis Assay:** Cells were seeded in 96-well plates (3 × 10^2^ cells/cm^2^). After 24 hours, it was ascertained that they had reached approximately 80% confluence, and the medium was replaced with an osteogenic differentiation medium (MSCgo OsteogenicXF, Biological Industries), in which they were incubated for 21 days. Subsequently, they were fixed in 70% ethanol at 4°C for 1 hour. They were then stained for 10 minutes at room temperature with filtered alizarin red solution to visualize mineral deposition.

**Chondrogenesis Assay:** Cells were seeded in 96-well plates (3 × 10^2^ cells/cm^2^). After 24 hours, it was ascertained that they had reached approximately 80% confluence, and the medium was replaced with a chondrogenic differentiation medium (MSCgo ChondrogenicXF, Biological Industries, Beit Haemek, Israel), in which they were incubated for 21 days. Subsequently, Dulbecco’s phosphate-buffered saline (DPBS) was carefully removed, and the cells were fixed in 4% formaldehyde and incubated at room temperature for 30 minutes. The proteoglycan protein aggrecan was blue-stained using Alcian blue staining.

## Microcarrier Cultivation

### We used the following 2 types of microcarriers

**Hillex II (Solohill, PALL Corporation, Port Washington, NY, USA):** Microcarriers have an amine-treated surface and consist of particles with densities of 1090-1150 kg/m^3^ for sizes of 160-200 µm.

**Pronectin F (Solohill, PALL Corporation, Port Washington, NY, USA):** These are styrene-coated microcarriers with densities of 1022-1030 kg/m^3^ and particle sizes of 125-212 µm.

Cell cultures were made with microcarrier in a treated (positively charged) 60-mm-diameter culture plate and an untreated characterized 60-mm-diameter IVF culture plate, both stem cell adhesion amounts were compared to the culture plate. The microcarriers were weighed with precision scales to provide a surface area of 10 cm^2^/mL and added to 60-mm cell culture dishes. Mesenchymal stem cell of 3000 cells/cm^2^ was cultivated by calculating the total surface area of the microcarrier and culture dish.

Dulbecco’s modified Eagle medium F-12 basal medium prepared with 7.5% platelet lysate, penicillin–streptomycin, and l-glutamine additives was used for the proliferation of cells. An orbital shaker (Nüve SL-350) rotating at 50 turns/min was operated for 10 min/h. Cells were cultured in this manner for 24 hours.

## Groups

**Figure 2. A-C.**
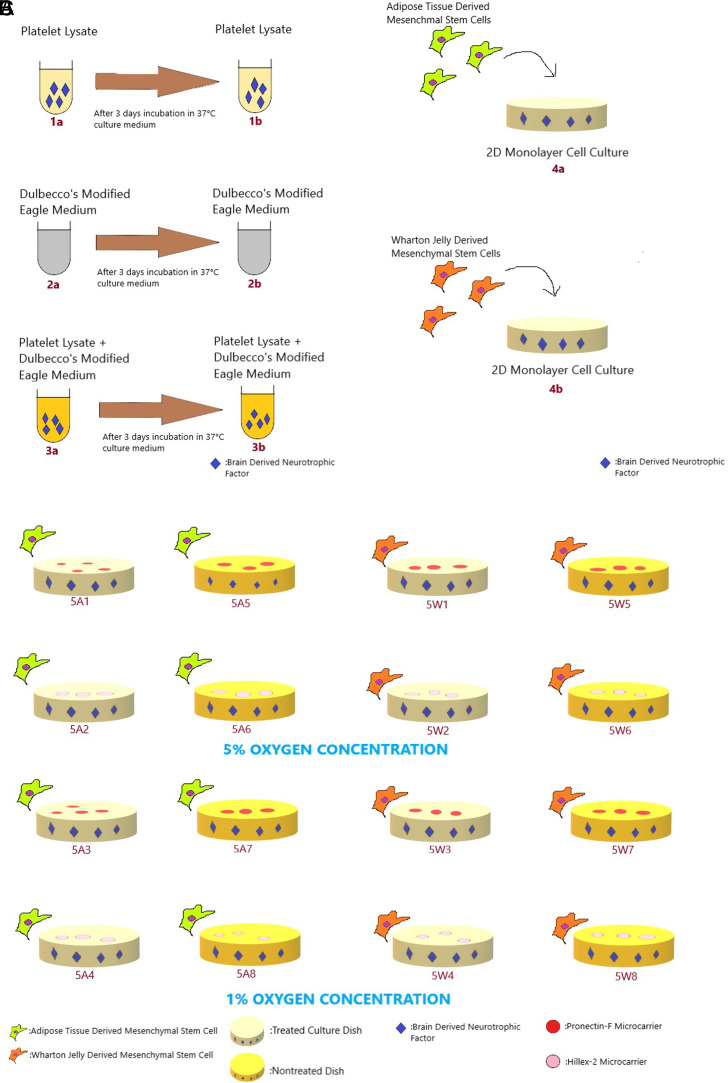
Amounts of BDNF were measured in each group in pictures A, B, and C (1a, 1b, 2a, 2b, 3a, 3b, 4a, 4b, 5A1, 5A2, 5A3, 5A4, 5A5, 5A6, 5A7, 5A8, 5W1, 5W2, 5W3, 5W4, 5W5, 5W6, 5W7, 5W8). BDNF, brain-derived neurotrophic factor.

1. BDNF measurement in pure lysate:
  - Freshly dissolved (1/1, 1/2, 1/3, 20/980, 10/990),
  - After waiting for 3 days at 37°C culture conditions, to determine whether there is a decrease in BDNF level in lysate (1/1, 1/2, 1/3, 20/980, 10/990).
2. BDNF measurement in pure MEM: 2a) freshly dissolved (1/1, 1/2, 1/3), 2b) after waiting for 3 days at 37°C culture condition BDNF measurement (1/1, 1/2, 1/3).
3. MEM with 7.5% lysate added: 3a) freshly dissolved (1/1, 1/2, 1/3), 3b) after waiting for 3 days at 37°C in culture conditions BDNF measurement (1/1, 1/2, 1/3).
4. 2D monolayer cell culture: 4a) adipose tissue-derived stem cell 2D monolayer cell culture, 4b) Wharton’s jelly-derived 2D monolayer cell culture (Figure 2).
  In the first part of our study, we completed the first 4 groups as described above. Our fifth group was comprised of 3D microcarrier cell cultures. In our study, we made cell cultures using 2 different percentages of O_2_ (1% and 5%), 2 different types of microcarriers (hillex and pronectin), 2 different sources of MSC (adipose-derived stem cell (ADSC) and Wharton’s jelly-derived stem cell (WJDSC)), and 2 different culture dishes (cell culture dish and IVF dish). We observed and photographed cell adhesions to microcarriers. We measured BDNF levels in 16 different groups via ELISA on the sixth day in cell culture fluids:
5. 3D microcarrier cell culture:

ADSC groups: 5A1. ADSC-culture dish (CD)-5%-pronectin (P), 5A2. ADSC-CD-5%- Hillex (H), 5A3. ADSC-CD-1%-P, 5A4. ADSC-CD-1%-H, 5A5. ADSC- in vitro fertilization dish (IVFD)-5%-P, 5A6. ADSC-IVFD-5%-H, 5A7. ADSC-IVFD-1%-P, 5A8. ADSC-IVFD-1%-H.

WJDSC groups: 5W1. WJDSC-CD-5%-P, 5W2. WJDSC-CD-5%-H, 5W3. WJDSC-CD-1%-P, 5W4. WJDSC-CD-1%-H, 5W5. WJDSC-IVFD-5%-P, 5W6. WJDSC-IVFD-5%-H, 5W7. WJDSC-IVFD-1%-P, 5W8. WJDSC-IVFD-1%-H.

### Brain-Derived Neurotrophic Factor Enzyme-Linked Immunosorbent Assay

Brain-derived neurotrophic factor levels of cell culture supernatants were measured using ELISA kits (RayBio Human BDNF ELISA Kit, RayBiotech Life, Peachtree Corners, GA, USA) according to the manufacturer’s instructions. The concentrations of BDNF were calculated by measuring the absorbance at 450 nm using a microplate reader (Elx800, BIO-TEK Instruments, Inc., Winooski, VT, USA). All standards or samples were run in duplicate.

**Figure 3.**
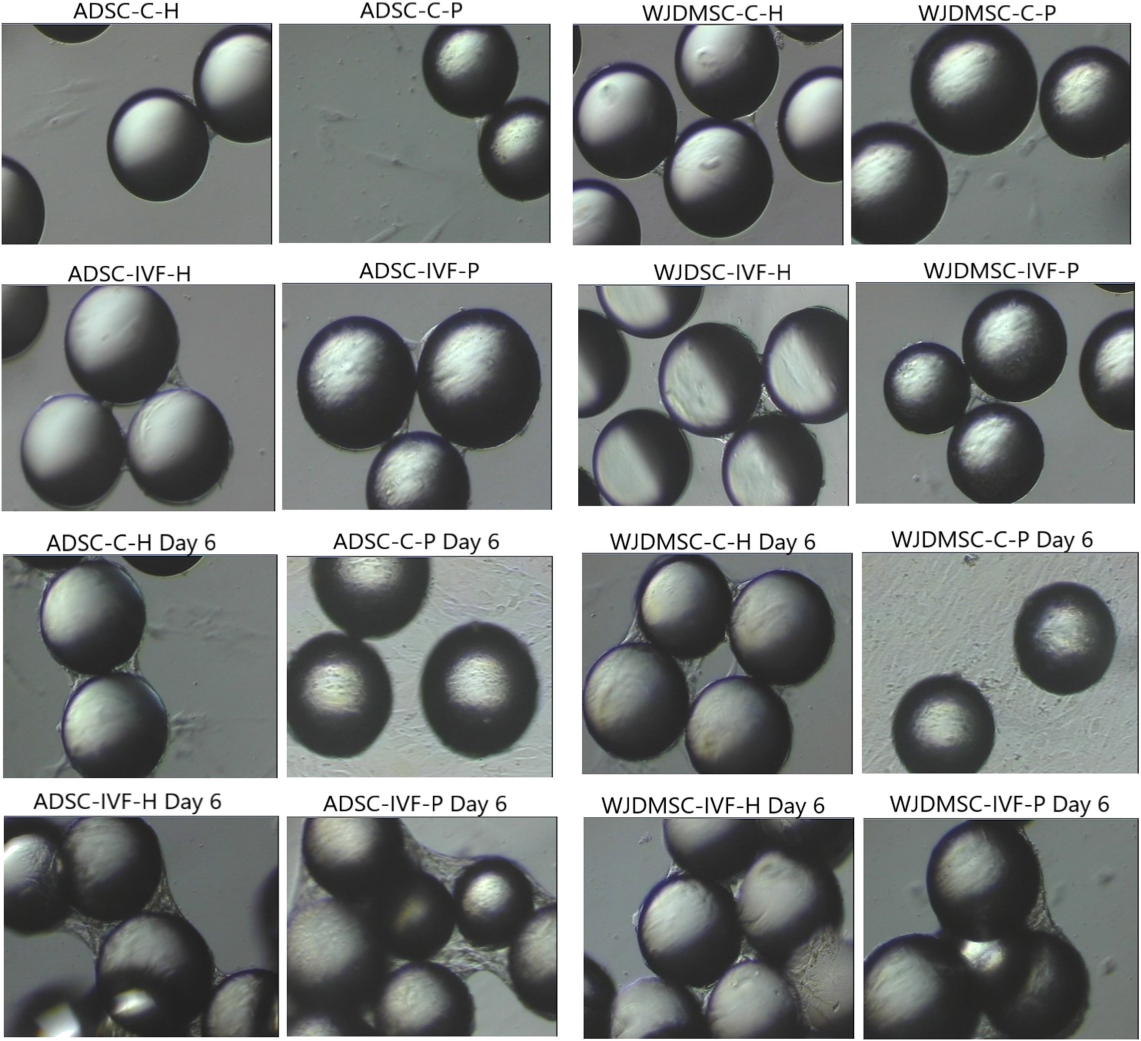
ADSC and WJDSC microcarrier cultivation. ADSC, adipose-derived stem cell; WJDSC, Wharton’s jelly-derived stem cell.

To create control group, we kept the culture medium prepared with 7.5% lysate for 3 days at 37°C without cultivation under the same culture conditions. Brain-derived neurotrophic factor level was also measured in culture medium containing only DMEM. Brain-derived neurotrophic factor measurement was also performed in pure lysate. In our study, we used MSCs from 2 different sources (ADSC and Wharton’s jelly). We used 2 different microcarriers (hillex–pronectin) to provide 3D cell culture (Figure 3). To compare, the BDNF level was measured in both cell groups in 2D (monolayer) culture. We evaluated BDNF levels in cultures made with different microcarriers in 1% and 5% O_2_ with hypoxia incubator chamber culture media in order to evaluate the oxygen level, which is important and is a current subject in the cell culture.

## Results

We kept the culture medium prepared with 7.5% lysate for 3 days at 37°C without cultivation under the same culture conditions. As a result, the BDNF level was the same as the first measured amount. There was no change in the amount of BDNF in the lysate culture medium, pure lysate, and pure medium at 37°C in culture conditions.

1. **BDNF measurement in pure lysate:**
  2,263,700 ng/mL,
  1,063,100 ng/mL,
2. **BDNF measurement in pure MEM:**
  0 ng/mL,
  0 ng/mL
3. **MEM with 7.5% lysate added:**
  27,234 ng/mL,
  28,332 ng/mL
4. **2D monolayer cell culture:**
  2,278 ng/mL,
  1,590 ng/mL
  When we cultured monolayer stem cells in the lysate medium, there was a decrease (2278 ng/mL) in the amount of BDNF present in the cell-free culture medium measurement (27 234 ng/mL).
5. **3D microcarrier cell culture:**
  - **5A1. **ADSC-CD-5%-P: 8346 ng/mL, **5A2. **ADSC-CD-5%-H: 4714 ng/mL, **5A3. **ADSC-CD-1%-P: 3516 ng/mL, **5A4. **ADSC-CD-1%-H: 5868 ng/mL, **5A5**. ADSC-IVFD-5%-P: 12,066 ng/mL, **5A6**. ADSC-IVFD-5%-H: 17,278 ng/mL, **5A7. **ADSC-IVFD-1%-P: 20,026 ng/mL, **5A8. **ADSC-IVFD-1%-H: 29,686 ng/mL,
  - **5W1. **WJDSC-CD-5%-P: 7408 ng/mL, **5W2. **WJDSC-CD-5%-H: 7336 ng/mL, **5W3**. WJDSC-CD-1%-P: 7284 ng/mL, **5W4**. WJDSC-CD-1%-H: 7562 ng/mL, **5W5. **WJDSC-IVFD-5%-P: 13,866 ng/mL, **5W6. **WJDSC-IVFD-5%-H: 16,716 ng/mL, **5W7. **WJDSC-IVFD-1%-P: 17,896 ng/mL, **5W8. **WJDSC-IVFD-1%-H: 20,188 ng/mL

**Figure 4.**
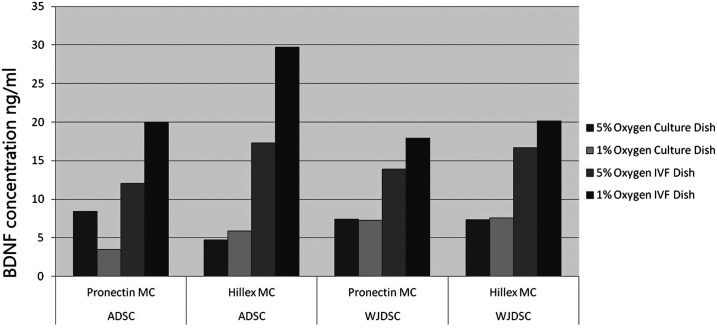
Graph of BDNF concentrations in ADSC and WJDSC hillex/pronectin microcarrier, 1%/5% oxygen and culture dish/in vitro fertilization dish groups. BDNF, brain-derived neurotrophic factor; ADSC, adipose-derived stem cell; WJDSC, Wharton’s jelly-derived stem cell.

The BDNF level in the lysated culture medium was significantly lower than the levels in 2D culture (Table 1). In the cultures made with the microcarrier, the sixth day of the culture (75%-80% occupancy) exhibited higher BDNF levels. Brain-derived neurotrophic factor levels were higher in the 1% O_2_ group. In our study, the amount of BDNF measured at a 1% O_2_ level was higher than the 5% O_2_ level in each group. The group with the highest BDNF level was the group with 1% O_2_ Hillex microcarrier (MC). A considerable difference in BDNF levels could not be inferred between ADSC and WJDSC cell groups(Figure 4).

## Discussion

In this article, we examined the large-diameter stem cell class (mesenchymal, originating from adipose tissue and Wharton jelly), which is important for the success of cell therapy in clinical studies. Both of these cell types are grown universally in 2D cultures. However, for large-scale production, bioreactors have a shift toward 3D suspension cultures, especially with the use of MCs.^18^

Large amounts of cells are needed for clinical treatments, and thousands of treatment doses can be difficult to produce in traditional monolayer culture. With the increasing demand, conventional labor becomes intense, costly, demanding, and impractical. One of the options to overcome this obstacle is MC-based culture technology. Culturing with MC can be performed with a spinner flask or plate-using shaker.^19^ Three-dimensional culture conditions stimulate the environment of cells in vivo, thereby providing a favorable condition that increases cellular activities not observed in normal monolayer cultures, which has been shown in studies.^20,21^ One study with microcarrier culture showed that the enhanced migration and secretion capabilities suggest that microcarrier culture may enhance hMSC therapeutic potential in immunomodulation, angiogenesis, and neural differentiation.^22^

Brain-derived neurotrophic factor, which is commonly found in many areas, such as the hypothalamus and cortical areas in the central nervous system, has shown to accumulate in platelets, vascular endothelium, neuromuscular synapse, and muscle and liver tissue for peripheral neuronal repair. It has been shown to have protective effects on ischemic brain injury.^23^ Brain-derived neurotrophic factor levels have been associated with many neurological diseases, such as Alzheimer’s, schizophrenia, and depression.^14,24^ The neuroprotective and therapeutic effects of MSCs together with BDNF have been demonstrated in optic nerve damage^25^ and neuron ventral root damage,^26^ axonal growth,^27^ Huntington's disease,^28^ and multiple sclerosis.^29^

Brain-derived neurotrophic factor is a protein commonly found in the mammalian brain and can be taken up in the cells and stored in vesicles.^30^ In studies, expressions showing the production of BDNF in cells in MSCs were detected.^31^ Brain-derived neurotrophic factor can be stored in human platelets, and the main source of peripheral BDNF is platelets.^14,32^ The amount of BDNF in the culture liquid we prepared with the addition of 7.5% platelet lysate did not change at the end of the sixth day in the same incubator at 37°C, the conditions in which we made cell culture. However, there was a decrease in BDNF level in ADSC and WJDSC monolayer cell culture, which we prepared with the addition of 7.5% lysate in the same volume liquid. In the next stage of our study, we wanted to evaluate whether we had achieved different results by changing the culture conditions. We used ADSC and WJDSC as the MSC source and aimed to increase cell adhesion to microcarriers using an untreated IVF dish. We kept the number of stem cells we added to the cell culture fluid consistent and worked under the same incubator culture conditions. We measured higher levels of BDNF in cultures where we used microcarriers. Similar BDNF levels were measured in 2 different cell sources, and when we reduced the O_2_ level to 1%, we reached the highest BDNF levels. In recent studies, it has been shown that MSCs increase paracrine abilities in hypoxic environments.^33,34^

The limitation of our study is that it has not been evaluated with a full intergroup analysis due to a large number of study groups. This also reduces the intelligibility of our study. Our aim was to address the issue with a general approach and to determine the points that need to be focused on in future studies and to be studied more specifically. In this case, it will be useful to evaluate the changes in culture with a single microcarrier by focusing on the oxygen level by including different growth factors.

In a different area of research, it is possible to study BDNF reuptake from stem cells. Recent studies have shown BDNF secretion from stem cells.^35-37^ However, we know that changes in culture conditions affect the behavior of stem cells. The dynamic culture microenvironment facilitates the flow of gases and nutrients and the removal of waste products from the cells. It is also known that current stress affects the behavioral characteristics of cells in cultures.^38^ As a result of our observations, we think that the cells can exhibit greater therapeutic potential in a dynamic adhesion environment.

## Figures and Tables

**Table 1.** Brain-Derived Neurotrophic Factor Levels in Mesenchymal Stem Cell Culture Fluid Under Different Culture Conditions

	**2D Monolayer** (ng/mL)	**3D Microcarrier** (ng/mL)	**O _2__,_** **5%**		**O _2 __,_ 1%**	
			**Culture Dish** **(ng/mL)**	**In Vitro Fertilization Dish** **(ng/mL)**	**Culture Dish** **(ng/mL)**	**In Vitro Fertilization Dish (ng/mL)**
Adipose tissue-derived stem cell	2278	Pronectin F microcarrier	8346	12,066	3516	20,026
		Hillex II microcarrier	4714	17,278	5868	29,686
Wharton’s jelly-derived stem cell	1590	Pronectin F microcarrier	7408	13,866	7284	17,896
		Hillex II microcarrier	7336	16,716	7562	20,188
